# TRAF3 regulates the oncogenic proteins Pim2 and c-Myc to restrain survival in normal and malignant B cells

**DOI:** 10.1038/s41598-019-49390-9

**Published:** 2019-09-09

**Authors:** Amy L. Whillock, Nurbek Mambetsariev, Wai W. Lin, Laura L. Stunz, Gail A. Bishop

**Affiliations:** 1 Department of Microbiology & Immunology, University of Iowa, Iowa City, IA USA; 2 Internal Medicine, University of Iowa, Iowa City, IA USA; 3 Immunology Graduate Program, University of Iowa, Iowa City, IA USA; 4 Medical Scientist Training Program, University of Iowa, Iowa City, IA USA; 5 Holden Comprehensive Cancer Center, University of Iowa, Iowa City, IA USA; 6 VA Medical Center, Iowa City, IA USA; 7 Present Address: Sanford-Burnham-Prebys Medical Discovery Institute, La Jolla, CA USA; 8 Present Address: Northwestern Memorial Hospital, Chicago, IL USA

**Keywords:** Signal transduction, Lymphoma

## Abstract

TRAF3 is a versatile intracellular adapter protein with multiple context-specific roles. Uniquely in B cells, TRAF3 deficiency enhances survival and increases the risk of transformation, as loss of TRAF3 is observed in several types of B cell cancers. Here, we report a new mechanism for TRAF3 in the restraint of B cell survival. We found that TRAF3 deficiency was associated with induction of the pro-survival kinase Pim2 in mouse primary B cells and human malignant B cell lines. The increase in Pim2 was independent of NF-κB2 activation but was ameliorated with inhibition of STAT3 expression or function. TRAF3 deficiency also led to a Pim2-dependent increase in c-Myc protein levels and was associated with reduced c-Myc ubiquitination. TRAF3-deficient primary B cells were less sensitive to cell death induced by the Pim inhibitors SGI-1776 and TP-3654. Interestingly, human malignant B cell lines with low expression of TRAF3 were more sensitive to Pim inhibition-induced cell death. Combination treatment of TRAF3-deficient B cells and B cell tumor lines with c-Myc inhibitors enhanced their sensitivity to Pim inhibition, suggesting a possible therapeutic strategy. TRAF3 thus suppresses a Pim2-mediated B cell survival axis, which can be a potential target for treatment of B cell malignancies.

## Introduction

The adaptor signaling protein tumor necrosis factor receptor (TNFR)-associated factor 3 (TRAF3) displays cell and context-specific regulation of a variety of signaling pathways, with distinct consequences in different cell types^1,2^. Whole-organism *Traf3* deletion in mice leads to neonatal death, demonstrating the critical roles played by TRAF3 in key biological functions^3^. When genetic loss of *Traf3* is restricted to the mouse B cell lineage (B-*Traf3*^−/−^ mice), these mice display a striking phenotype resulting from markedly enhanced B cell survival, including accumulation of B cells in all secondary lymphoid organs, as well as occurrence of spontaneous germinal centers and autoantibody production^4,5^. As B-*Traf3*^−/−^ mice age, most develop B cell lymphoma, consistent with the prediction that their enhanced B cell survival allows accumulation of mutations that ultimately increase the likelihood of transformation^6^. Importantly, genetic loss of *TRAF3* in humans is also associated with B cell malignancies. It has been reported that >15% of diffuse large B cell lymphomas (DLBCL) and ~20% of multiple myelomas contain loss and/or loss-of-function mutations in *TRAF3*^7–10^. Additionally, TRAF3 deficiency in human B cell tumors may result from post-translational modification. We recently showed that the Epstein-Barr virus (EBV)-encoded oncogenic protein latent membrane protein 1 (LMP1), through its high-avidity binding of TRAF3, can sequester TRAF3 away from regulating other pro-survival pathways in the cell, producing a TRAF3-deficient phenotype in mouse and human DLBCL cell lines^11^. TRAF3 is therefore an important B cell tumor suppressor.

The TNF family member B cell activating factor (BAFF) is critical to the survival of normal B cells. A critical part of signaling through the BAFF receptor (BAFFR) is activation of the serine-threonine kinase NF-κB inducing kinase (NIK). NIK plays a key role in activation of the pro-survival non-canonical NF-κB2 pathway. TRAF3 normally restrains NIK activation, as part of a cytoplasmic complex of proteins that mediate NIK poly-ubiquitination and proteasome-mediated degradation. BAFFR signaling recruits this complex to the membrane, after which TRAF3 itself becomes poly-ubiquitinated and degraded^12,13^. B cells lacking TRAF3 no longer require BAFF signals to survive, and display constitutive nuclear localization of NF-κB2 factors p52 and RelB^2^. However, it was subsequently discovered that T cells, dendritic cells, and macrophages lacking TRAF3 also display constitutive NF-κB2 activation, but show no increases in cellular survival^14,15^. In B cells, degradation of TRAF3 is also neither sufficient nor required for the activation of the NF-κB2 pathway^16^. Thus, TRAF3 regulates B cell survival via multiple mechanisms, which include inhibition of transcription of the pro-survival protein Mcl-1^17^, and restraint of B cell glucose metabolism^18^.

Prior gene expression analysis compared normal and TRAF3-deficient B cells versus WT and TRAF3-deficient T cells to identify TRAF3-regulated targets in B cells associated with their uniquely enhanced survival^17^. This analysis revealed an increase in the kinase proviral insertion in murine lymphoma (Pim) 2 in TRAF3^−/−^ B cells. The Pims are a transcriptionally-regulated, constitutively active serine/threonine kinase family consisting of Pim1, Pim2 and Pim3^19^. Pim2 is overexpressed in human MM cells and is required for their proliferation and survival^20,21^. Pim2 inhibition also promotes death in B lymphoma cells^22^.

We show here that loss of TRAF3 was sufficient to induce Pim2 up-regulation in B cells. This increase was not attenuated by deletion of NIK. TRAF3-deficient B cells were more susceptible to biochemical Pim2 inhibition and more resistant to inhibitors of the phosphatidyl inositol-3 kinase/protein kinase B (PI3K/Akt) pathway compared to wild type (WT) B cells. Low TRAF3 expression in human BCL and MM cell lines correlated with higher Pim2 protein levels and decreased susceptibility to Pim2 inhibitor-mediated cell death. Interestingly, B cell TRAF3 deficiency also resulted in Pim2-dependent elevation in c-Myc protein levels without a change in c-Myc mRNA; this was associated with decreased c-Myc poly-ubiquitination. B cells lacking TRAF3 were resistant to inhibition of c-Myc expression but were susceptible to inhibition of c-Myc activity. Inhibition of c-Myc also enhanced cell death in response to a Pim2 inhibitor. These findings identify Pim2 as an important mediator of enhanced survival in B cells that coordinates with several related pro-viability molecular mechanisms. Pim2-regulated pathways thus present a promising potential therapeutic target in TRAF3-deficient B cell malignancies.

## Results

### TRAF3-mediated regulation of B cell Pim2 expression

**Figure 1:**
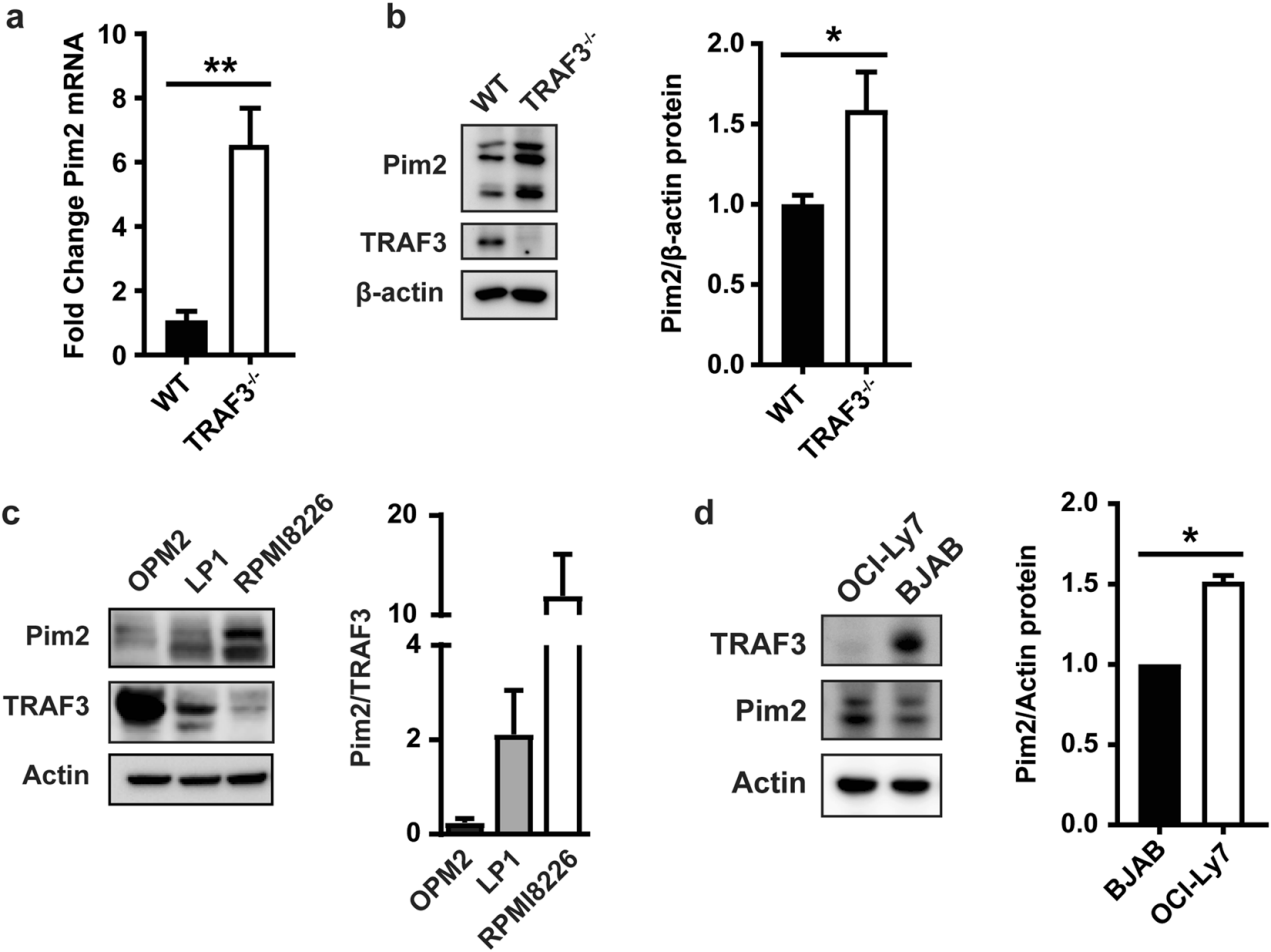
TRAF3-mediated regulation of Pim2 expression in mouse primary B cells and human MM and BCL cell lines. (**a**) Pim2 mRNA levels in WT and TRAF3^−/−^ B cells were determined by RT-PCR. Data were normalized to GAPDH and fold change was determined using the comparative Ct method. Graph depicts mean values ± SEM (N = 3 mice). An unpaired t test was used to evaluate differences for statistical significance (**p < 0.01). (**b**) Whole-cell lysates (WCLs) of WT and TRAF3^−/−^ B cells were analyzed with Western blotting (WB) for protein expression. Graphs depict mean values ± SEM with (N = 8 mice from 2 independent experiments). Samples were normalized first to the β-actin loading control and then to the average WT normalized value. An unpaired t test with Welch’s correction was used to evaluate differences for statistical significance (*p < 0.05). (**c**,**d**) Relative levels of TRAF3 and Pim2 in indicated human MM (**c**) and DLBCL (**d**) cell lines were determined with WB. Representative blots from 3 (**c**) and 6 (**d**) independent experiments are shown. Graph in (**c**) represents relative levels of Pim2/actin divided by TRAF3/actin of the indicated MM cell lines (N = 3). Graph in (**d**) depicts mean values ± SEM. (**c**,**d**) were previously presented in the doctoral dissertation of N.M^23^. Wilcoxon signed rank test was used to evaluate differences for statistical significance (*p < 0.05; N = 6).

To determine the pathways involved in the hyper-survival of TRAF3-deficient B cells (TRAF3^−/−^), we performed a microarray analysis, comparing the gene expression of TRAF3^−/−^ mouse B cells compared to those of wild type littermate controls (WT)^17^. We also compared gene expression in TRAF3^−/−^ B cells versus TRAF3^−/−^ T cells from conditional knockout mice, as these T cells do not have enhanced survival^1,14^. *Pim2* gene expression was increased in TRAF3^−/−^ B cells compared to either WT B cells or TRAF3^−/−^ T cells. Confirming microarray data, TRAF3^−/−^ B cells had 6-fold higher expression of *Pim2* mRNA compared to WT B cells when examined by RT-PCR (Fig. 1a). Pim2 protein was also increased in TRAF3^−/−^ compared to WT B cells (Fig. 1b). Interestingly, TRAF3 deficiency specifically regulated the Pim2 isoform, as expression of Pim1 and Pim3 was unchanged (Supplemental Fig. 1).

Our observations in mouse primary B cells led us to predict that TRAF3 protein levels in B cell tumors would impact their relative levels of Pim2 protein. We examined 3 human MM-derived cell lines (OPM2, LP1, and RPMI8226) and observed an inverse correlation between their relative TRAF3 and Pim2 protein levels (Fig. 1c). In DLBCL-derived human cell lines, OCI-Ly7 cells had undetectable TRAF3 protein and increased Pim2 expression compared to TRAF3-positive BJAB cells (Fig. 1d). Figure 1c,d were previously presented in the doctoral dissertation of N.M.^23^. Although we expect that there are multiple gene alterations in tumor cells that could impact Pim2 expression, our results indicate that TRAF3 likely serves as an important regulator to restrain Pim2 expression at both the mRNA and protein levels in normal and malignant B cells. This conclusion is strengthened by the recent complementary finding described in the Introduction that human BCL cell lines expressing LMP1, which avidly binds and sequesters TRAF3, also display a TRAF3-deficient phenotype, including elevated Pim2 protein^10^.

### Effect of loss of TRAF3 on Pim2 target phosphorylation

**Figure 2:**
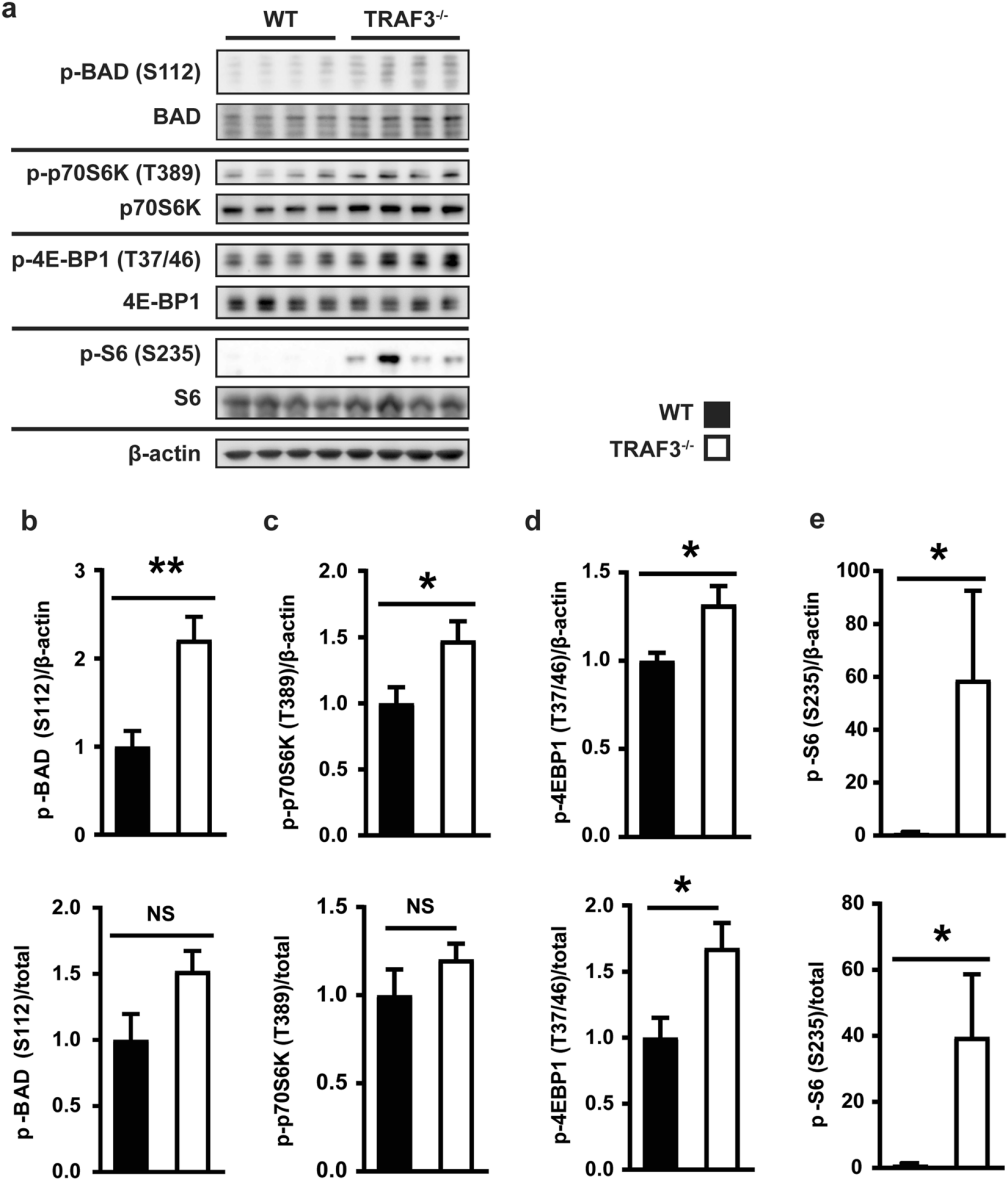
Effect of loss of TRAF3 on Pim2 target phosphorylation. (**a**) Whole cell lysates of WT and TRAF3^−/−^ B cells were analyzed with WB for protein expression. Each lane represents an individual mouse. (**b**–**e**) Relative levels of p-BAD S112, p-p70S6K T389, p-4E-BP1 T37/46, or p-S6 ribosomal protein S235 were normalized first to the β-actin loading control (top row) or total BAD, p70S6K, 4E-BP1, or S6 (bottom row) and then to the average normalized WT value. Graphs depict mean values ± SEM. Unpaired t test was used in (**b**–**d**) and Mann Whitney test was used in (**e**) to evaluate differences for statistical significance (*p < 0.05, **p < 0.01; N = 4).

Phosphorylation of the pro-apoptotic BAD protein at serine-112 by Pim2 inhibits cell death^24^. The kinase p70-S6 (p70-S6K), S6 ribosomal protein (S6), and 4E-BP1, involved in protein translation, are also Pim2 phosphorylation targets, and contribute to regulation of cell survival^25,26^. Increased expression of Pim2, which is a constitutively active kinase, within TRAF3^−/−^ mouse B cells resulted in enhanced expression of its known targets BAD^27^, p70-S6K, 4E-BP1, and ribosomal protein S6 (Fig. 2a–e), as well as phosphorylated (active) forms of these proteins. In the case of 4EBP1 and S6, there was also a selective increase in the phosphorylated forms, above the increase in total amounts. These results indicate that the increased Pim2 protein is accompanied by increased Pim2 activity in TRAF3-deficient B cells.

### Impact of TRAF3 and Pim2 upon c-Myc stability and levels

**Figure 3:**
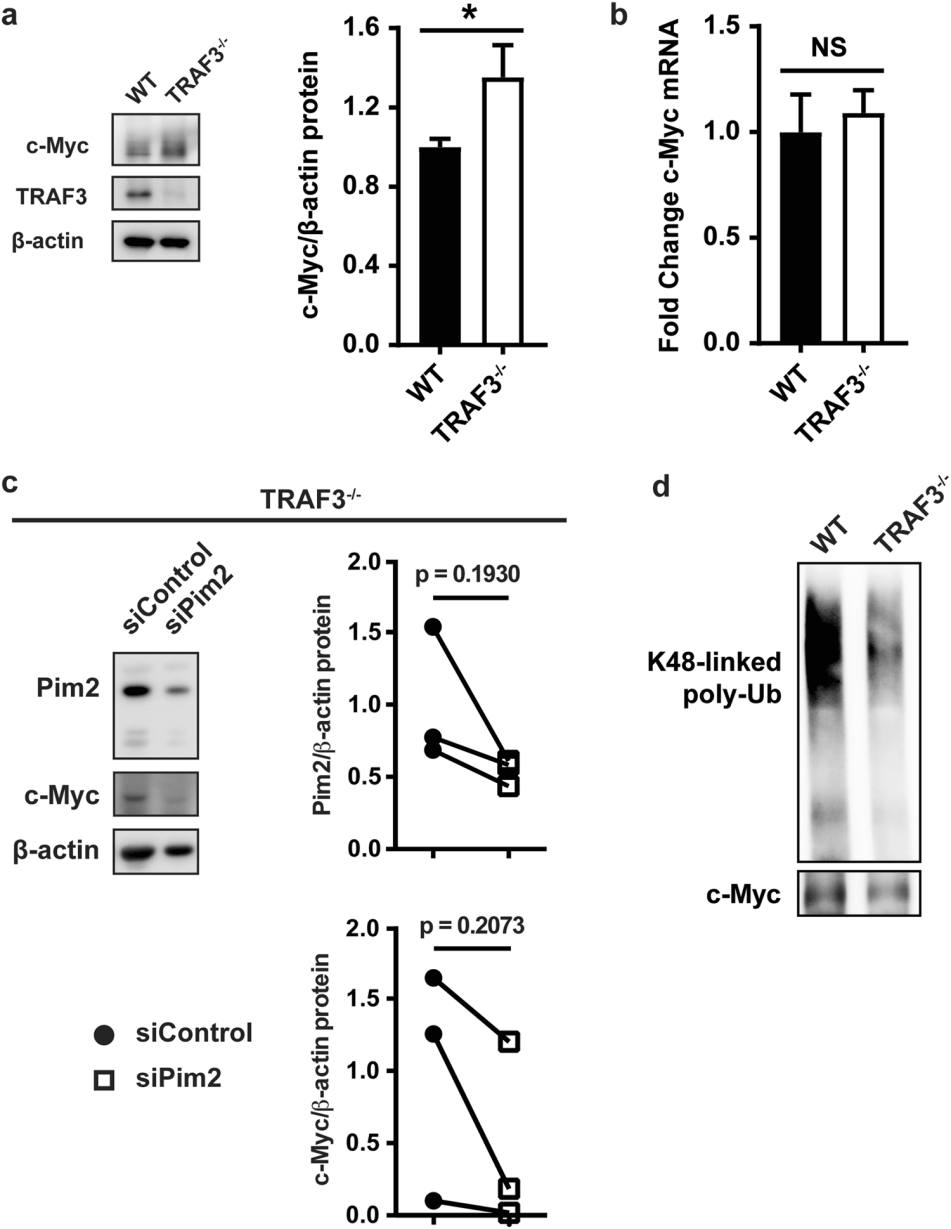
Pim2 regulation of c-Myc stability in the absence of TRAF3. (**a**) WCLs of WT and TRAF3^−/−^ B cells were analyzed with WB for c-Myc expression. Graph depicts mean values ± SEM (N = 11 from 3 independent experiments). An unpaired t test was used to evaluate differences for statistical significance (*p < 0.05). (**b**) c-Myc mRNA levels in WT and TRAF3^−/−^ B cells were assayed with RT-PCR. Data were normalized to GAPDH and fold change was determined using the comparative Ct method. Graph represents mean values ± SEM shown (N = 3 mice). Student’s t test was used to evaluate differences for statistical significance. (NS = not significant). (**c**) TRAF3^−/−^ B cells were transfected with siRNA scrambled control or siRNA targeting Pim2. 24 hrs later, Pim2 and c-Myc expression in WCLs were analyzed with WB. Graphs depict Pim2 or c-Myc expression/β-actin loading control and normalized to the siRNA control. A paired t test was used to evaluate differences for statistical significance (N = 3). (**d**) Following immunoprecipitation of c-Myc from WT and TRAF3^−/−^ B cells, K48-linked polyubiquitination was assayed with WB. Blot is representative of 3 independent experiments. (**d**) was previously presented in the doctoral dissertation of N.M^23^.

The transcription factor c-Myc has frequently been implicated in B cell oncogenesis^28,29^, and is a potential therapeutic target. Additionally, proviral tagging in *myc* transgenic mice revealed Pim2 as a collaborating oncogene driving lymphomagenesis^30^. Pim2 directly phosphorylates c-Myc on serine 329, leading to stabilization of c-Myc protein and enhancing cell transformation^31^. We thus investigated whether TRAF3-deficient B cells also display alterations in c-Myc expression and/or post-translational modification. TRAF3-deficient B cells had increased expression of c-Myc protein, without an increase in c-Myc mRNA (Fig. 3a,b). Inhibiting Pim2 expression with siRNA attenuated levels of c-Myc expression in TRAF3^−/−^ B cells (Fig. 3c). Although inter-mouse variability in absolute levels of both Pim2 and c-Myc resulted in differences that together did not reach statistical significance, the clear downward trend in c-Myc when Pim2 expression was reduced was consistent in all samples. Stability of c-Myc protein is regulated by K48-linked poly-ubiquitination^32^. In the absence of TRAF3, the elevated c-Myc protein levels were associated with reduction in K48-linked poly-ubiquitination of c-Myc (Fig. 3d; previously presented in the doctoral dissertation of N.M.^23^). These findings indicate that loss of TRAF3 in B cells results in increased cellular c-Myc protein, which requires the enhanced Pim2 expression downstream of TRAF3 deficiency and is associated with reduced c-Myc K48-linked ubiquitination.

To determine whether this increase in Pim2 was B cell-specific we examined T cell isolated from T cell-specific TRAF3^−/−^ mice^14^. There was no significant difference in Pim2 mRNA in TRAF3^−/−^ and WT T cells in microarray analysis^17^. Interestingly, Pim2 protein expression was increased in TRAF3^−/−^ T cells compared to WT T cells, although this difference was not statistically significant. There was no difference in c-Myc protein expression by T cells, related to TRAF3 status (Supplemental Fig. 2).

### STAT3 dependence of increased Pim2 in TRAF3-deficient B cells

As mentioned above, TRAF3 promotes degradation of NIK. In the absence of TRAF3, NIK accumulates and promotes activation of the NF-κB2 pathway (reviewed in^1^). B cell-specific deletion of NIK substantially decreases overall B cell survival and inhibits NF-κB2 activation^33^. Because BAFF-mediated induction of Pim2 is NF-κB2-dependent^34^, we tested the requirement for NIK in driving increased Pim2 expression of TRAF3-deficient B cells. Interestingly, loss of NIK in TRAF3-deficient B cells did not affect the elevated Pim2 (Supplemental Fig. 3; previously presented in the doctoral dissertation of N.M.^23^). Previously, it has been shown that TRAF3-deficient B cells still show enhanced survival when NIK is deleted^35^, suggesting that there are NIK-independent, pro-survival pathways that are regulated by TRAF3.

**Figure 4:**
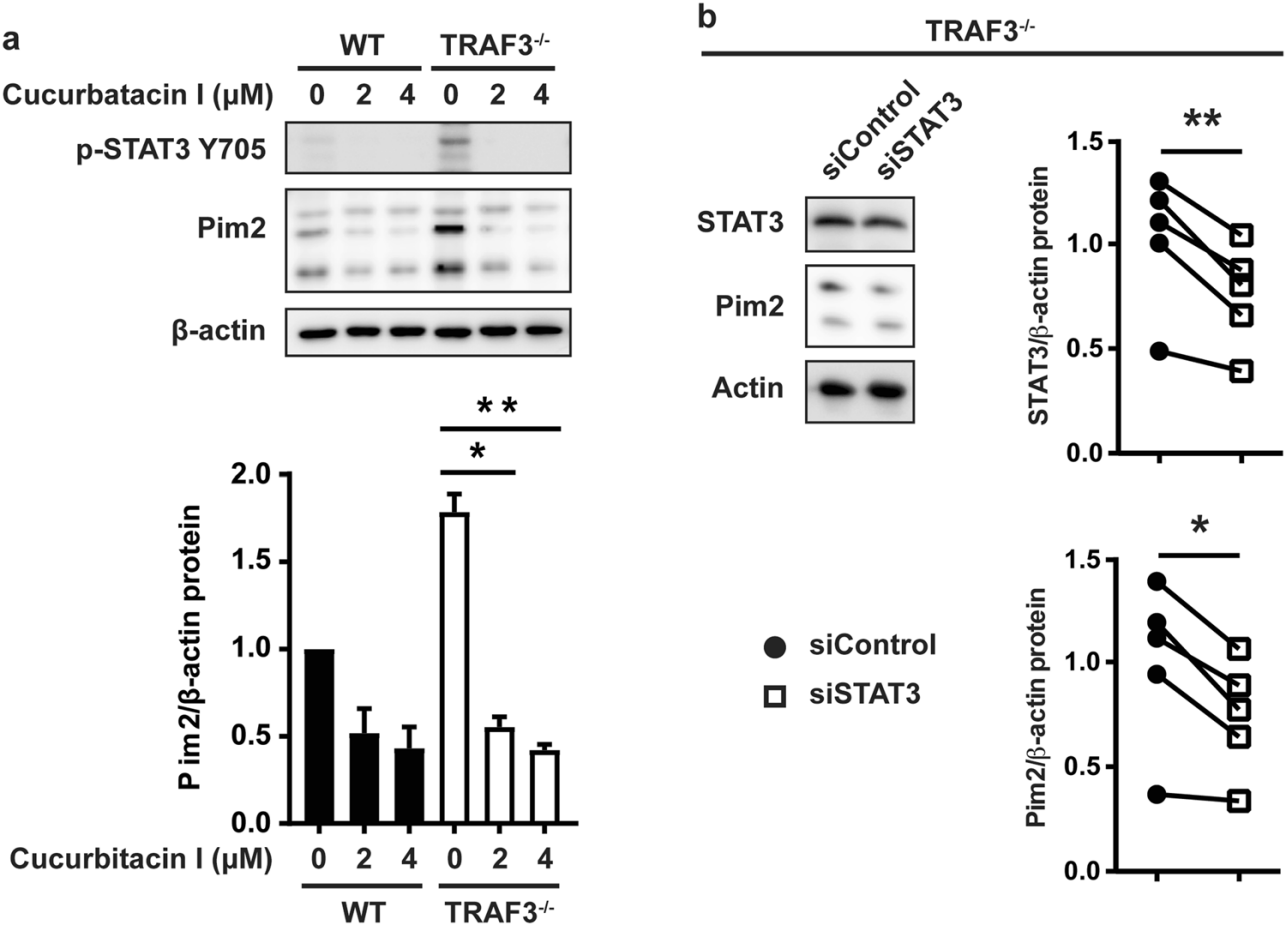
Impact of STAT3 inhibition in TRAF3-deficient B cells. (**a**) TRAF3^−/−^ B cells were treated with the indicated concentrations of the STAT3 inhibitor Cucurbatacin I for 16 hrs. Expression of p-STAT3 Y705 and Pim2 was analyzed by WB from WCLs. Blot is representative of 3 independent experiments. Pim2/β-actin loading control was normalized to the WT value. Graph shows mean values ± SEM shown (N = 3). An unpaired t test was used to evaluate differences for statistical significance (*p < 0.05, **p < 0.01). (**b**) TRAF3^−/−^ B cells were transfected with control or siRNA targeting STAT3. 24 hrs later, Pim2 and STAT3 expression in WCLs were analyzed by WB. Graphs depict STAT3 or Pim2 expression/β-actin loading control and normalized to the average of the siRNA control. A paired t test was used to evaluate differences for statistical significance (*p < 0.05, **p < 0.01, N = 5).

STAT3 activation increases Pim2 expression in both hematological and solid tumors^21,36,37^. TRAF3 inhibits STAT3 activation downstream of the IL-6 receptor by recruiting the phosphatase PTPN22^38^. We thus investigated the relationship between increased expression of Pim2 in TRAF3^−/−^ B cells and levels of STAT3 activation. After treatment with the STAT3 inhibitor Cucurbatacin I^39^, Pim2 protein in TRAF3^−/−^ B cells decreased to WT levels (Fig. 4a). Partial inhibition of STAT3 expression with siRNA in TRAF3^−/−^ B cells resulted in a similar decrease in Pim2 protein (Fig. 4b). Our earlier studies demonstrated that TRAF3 associates with the IL-6R following IL-6 binding, and recruits PTPN22 to this receptor to reduce IL-6-mediated STAT3 phosphorylation. However, IL-6 treatment did not further increase Pim2 levels in TRAF3^−/−^ B cells (data not shown), indicating that TRAF3 does not regulate Pim2-mediated homeostatic survival via the IL-6R pathway. Our results show that TRAF3 deficiency sustains increased Pim2 expression downstream of STAT3 activation and independently of NF-κB2 activation.

### Impact of TRAF3 status on susceptibility of B cells to Pim inhibitors

**Figure 5:**
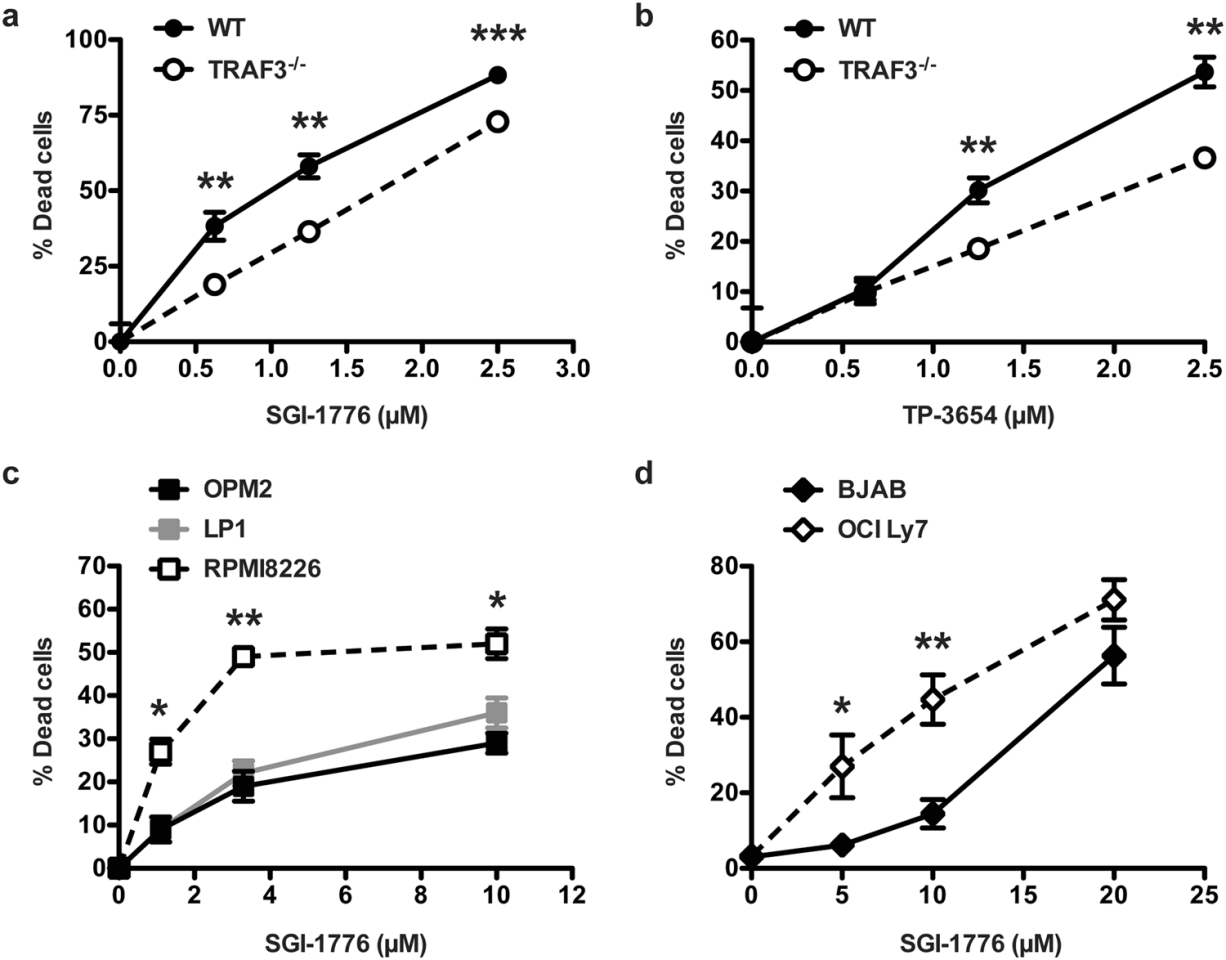
Effect of TRAF3 on susceptibility to kinase inhibition in mouse primary B cells and human MM and DLBCL cell lines. (**a**,**b**) Splenic B cells at 1 × 10^6^ B cells per well were treated with indicated doses of Pim inhibitors for 24 hrs. The viability was determined by PI exclusion. The percent viability was calculated as the mean viability of inhibitor-treated samples normalized to DMSO vehicle-treated sample for each genotype. Graphs indicate mean values ± SEM of percent viable cells treated with SGI-1776 (**a**) or TP-3654 (**b**). Graphs are representative of 3 independent experiments (N = 4). An unpaired Student’s t test was used to evaluate differences for statistical significance (*p < 0.05, **p < 0.01). (**c**,**d**) MM (**c**) and DLBCL (**d**) cell lines (5 × 10^3^/well) were treated with indicated doses of SGI-1776 for 24 hrs. The viability was determined by PI exclusion. Graphs indicate mean ± SEM of percent viability for cells normalized to the DMSO vehicle control. An unpaired Student’s t test was used to evaluate differences for statistical significance from 3 (**c**) or 7 (**d**) independent experiments (*p < 0.05, **p < 0.01, ***p < 0.001).

Pim2 is important for B cell survival, and has been suggested as a potential target for treatment of B cell malignancies^22,27^. To determine whether Pim2 inhibition induces death of TRAF3-deficient B cells, we tested B cell survival after 24 hr treatment with two different Pim kinase inhibitors: SGI-1776^40^ and TP-3654^41^. While isoform-restricted Pim2 inhibitors are not currently available, Supp. Fig. 1 indicates that the expression of other Pim isoforms is not increased in TRAF3-deficient B cells. Pim inhibition in both WT and TRAF3^−/−^ B cells led to increased cell death, consistent with the importance of Pim2 in B cell survival (Fig. 5a,b). TRAF3^−/−^ primary B cells were relatively more resistant to cell death mediated by both inhibitors, consistent with their elevated levels of Pim2 kinase.

We next wished to examine the relative sensitivity to Pim2 inhibition of human tumor-derived B cell lines with varying levels of TRAF3 protein (Fig. 1). While primary TRAF3^−/−^ B cells with elevated Pim2 resisted such inhibition, it is well-documented that tumors can develop dependence upon abnormally elevated proteins that promote growth and survival, a phenomenon known as ‘oncogene addiction’^42^. Consistent with such dependence, the TRAF3^low^ RPMI8226 MM cell line was more susceptible to Pim2 inhibition compared to higher TRAF3-expressing LP1 and OPM2 MM cell lines, and at lower inhibitor concentrations (Fig. 5c,d). TRAF3-deficient OCI-Ly7 BCL cells were also more susceptible to SGI-1776-induced death than TRAF3^high^ BJAB BCL cells at intermediate inhibitor concentration (Fig. 5d).

### Increased effect of combined inhibition of c-Myc and Pim2 on survival of TRAF3-deficient B cells

**Figure 6:**
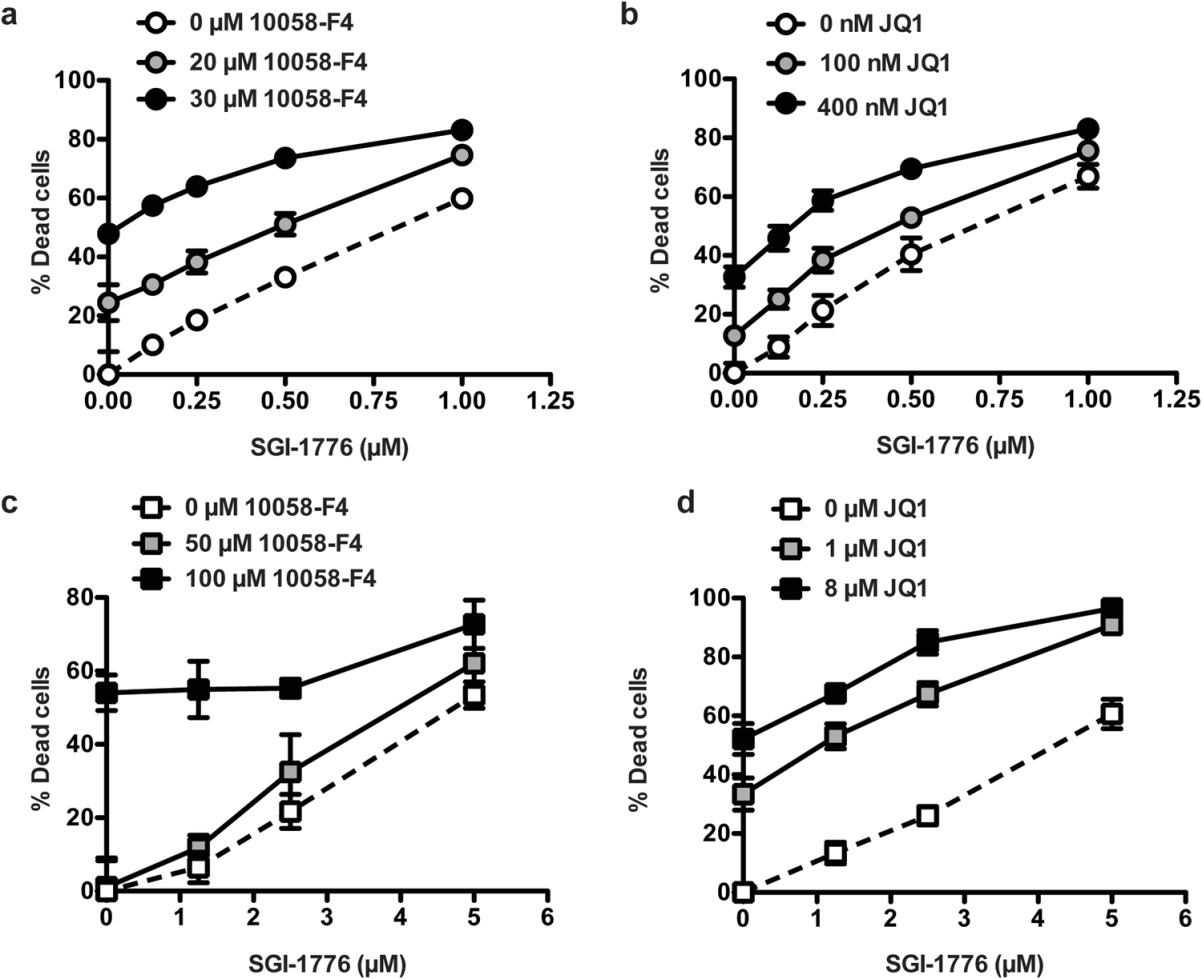
Impact of combined inhibition of c-Myc and Pim2 on survival of TRAF3-deficient B cells. (**a**,**b**) TRAF3^−/−^ splenic B cells at 1 × 10^6^ B cells per well were treated with the indicated combinations or SGI-1776 with JQ1 10058-F4 for 24 hrs. (**c**,**d**) The human MM-derivRPMI8226 cell line (1 × 10^5^ cells/well) was treated with the indicated combinations of SGI-1776 with JQ1 10058-F4 for 24 hrs. Viability was determined using PI exclusion and normalized to the DMSO vehicle control. Graphs depict the mean ± SEM of percent viability for cells (N = 3).

Inhibiting c-Myc expression is a potential therapeutic strategy to treat c-Myc-driven malignancies; c-Myc is the most frequently dysregulated gene in MM^43,44^. In DLBCL, 5% to 14% of tumors have c-Myc rearrangements or gene amplifications^45^. The drug JQ1 inhibits bromodomain-containing proteins, as these can promote c-Myc gene expression, and has shown promise in treatment of human MM and DLBCL^46,47^. To test whether targeting Myc can decrease the enhanced survival of TRAF3-deficient B cells, we treated WT or TRAF3^−/−^ B cells with the c-Myc inhibitors JQ1 or 10058-F4, the latter being an inhibitor of c-Myc-mediated transcriptional activity^48^. Combination treatment with either of the c-Myc inhibitors enhanced killing by the Pim inhibitor SGI-1776 in both TRAF3^−/−^ mouse primary B cells (Fig. 6a,b) and in the TRAF3-deficient human MM cell line RPMI8226 (Fig. 6c,d). As use of Pim inhibitors has been limited by their cardiotoxic effects at higher doses^41^, these results suggest that combined targeting of Pim2 and c-Myc expression may be beneficial, and allow lower doses of Pim inhibitors to be efficacious.

## Discussion

After it was revealed that TRAF3 plays a powerful B cell-specific role in restraint of homeostatic survival^2,4^, it has become evident that this TRAF3 role renders it an important tumor suppressor for B cell malignancies, particularly MM and BCL^9^. Because a striking component of the phenotype of all TRAF3-deficient cell types is constitutive activation of the NF-κB2 pathway^49^, it has often been assumed that enhanced NF-κB2 activation is both necessary and sufficient to explain how TRAF3 exerts its inhibition of B cell survival. However, a number of subsequent studies provide evidence that additional TRAF3-regulated pro-survival pathways, independent of NF-κB2, make important contributions to how TRAF3 restrains B cell survival (reviewed in 1, 2). We have thus worked to identify such pathways, as their characterization not only enhances understanding of basic TRAF3 functions, but also has important implications for choosing the most effective therapies for B cell malignancies.

**Figure 7:**
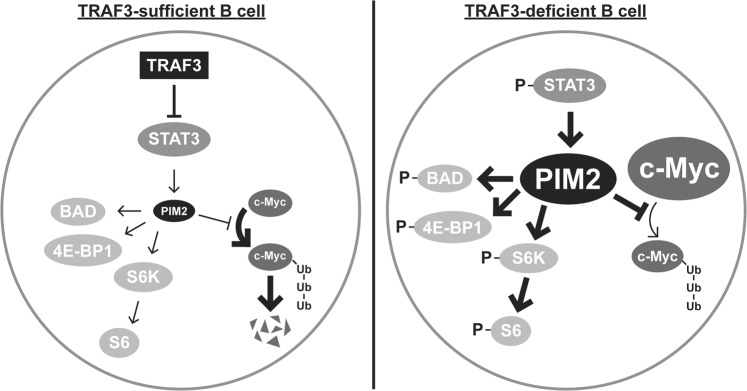
Model of Pim2 regulation in TRAF3-sufficient and –deficient B cells. In TRAF3-sufficient cells, TRAF3 inhibits STAT3 phosphorylation, resulting in decreased Pim2 protein. In the absence of TRAF3, there is increased STAT3 phosphorylation and Pim2 protein resulting in stabilization of c-Myc protein, and increased phosphorylation of the Pim2 targets BAD, p70S6K, 4E-BP1, and S6.

Our results presented here show that loss of B cell TRAF3 led to induction of the constitutively active pro-survival kinase Pim2, and enhanced expression and activation of its downstream targets BAD, p-70S6K, S6, and 4E-BP1 (Fig. 7). Interestingly, the Pim2 increase in TRAF3^−/−^ B cells is not attenuated with loss of NIK, suggesting that in these cells Pim2 levels are enhanced in TRAF3-deficient B cells independently of NF-κB2 activation. Our results also further illustrate multiple ways in which TRAF3 deficiency can enable and enhance pathogenesis of B cell malignancies. NF-κB2 is required for homeostasis of WT B cells^33^, but in TRAF3-deficient B cells certain oncogenic programming can be maintained independently of NF-κB2. This could potentially allow TRAF3-deficient B cells to serve as a reservoir for tumor recurrence and resistance to proliferation-targeting chemotherapy, as well as accumulation of additional oncogenic mutations.

TRAF3-deficient B cells maintained their elevated Pim2 expression in the absence of NIK, the initiating kinase of the NF-κB2 pathway, so we sought other upstream pathways that mediate the Pim2 increase resulting from TRAF3 deficiency. We observed here constitutive phosphorylation of STAT3 in TRAF3-deficient B cells and show that inhibition of STAT3 expression or function in TRAF3-deficient B cells decreased Pim2 elevation. Thus, TRAF3 in normal B cells inhibits Pim2 expression at least in part by restraining activation of STAT3, and by doing so inhibits B cell homeostatic survival, independent of direct engagement of the IL-6R. Interestingly, STAT3 is frequently constitutively active in bone marrow B cells from MM patients^50^, and TRAF3 deficiency is considered an early event in MM oncogenesis^51^.

Alternations in function of the oncogene *c-Myc* have long been implicated as playing an important role in the pathogenesis of BCL and MM^44^. Additionally, increased expression of c-Myc protein is associated with a poorer prognosis in patients with DLBCL, independent of other prognostic factors^45,52^. A substantial proportion (29% to 47%) of DLBCL have high protein expression of c-Myc, which, interestingly, is higher than the incidence of *c-Myc* gene translocations^45^. This suggests that in some cases of DLBCL, c-Myc protein is increased through alternative mechanisms such as post-translational modifications, and not purely from genetic alterations. c-Myc has many transcriptional targets and wide-ranging impacts on cell proliferation and growth. It is thus intriguing that the increase in Pim2 mediated by loss of TRAF3 also impacted B cell levels of c-Myc. Unlike the mRNA-directed increase in Pim2, this elevation in c-Myc was accomplished at the level of protein regulation, by decreased K48-linked poly-ubiquitination of c-Myc in TRAF3-deficient B cells, resulting in greater c-Myc protein stability. The present results are the first demonstration to our knowledge of Pim2-mediated enhancement of B cell c-Myc mediated at the post-translational level. In addition, c-Myc has pro-apoptotic functions^45^. Additional pro-survival pathways mediated by increased Pim2 expression, such as inhibition of activity of the pro-apoptotic BAD protein, may help to counteract the pro-apoptotic functions of c-Myc in TRAF3-deficient B cells, allowing their markedly enhanced viability.

In contrast to TRAF3-deficient B cells, TRAF3-deficient T cells do not have increased viability and loss of TRAF3 has not been reported to be associated with T cell malignancies^5^. TRAF3^−/−^ T cells do have constitutive NF-κB2 activation, a feature we and others have seen in all TRAF3-deficient cell types examined to date^4,5,15^. Here, we found that TRAF3^−/−^ T cells had a modestly trending increase in Pim2 protein. TRAF3^−/−^ T cells do not have an increase in STAT3 expression or activation, suggesting another mechanism is responsible for the increased Pim2 in these T cells^53^. One possible explanation is that TRAF3^−/−^ mice have increased regulatory T cells (Tregs) compared to WT mice^54^ and Tregs have increased Pim2 expression compared to conventional T cells, due to increased FoxP3 expression^55^. It would be interesting to explore this pathway further in future studies.

Our findings are summarized in a proposed model for TRAF3-mediated regulation of Pim2 and c-Myc in B cells (Fig. 7). These results have implications for selection of the most efficacious pathway inhibitors for treatment of TRAF3-deficient B cell malignancies, whether resulting from alterations in TRAF3 genes or post-translational protein reduction. Although TRAF3 levels inversely correlated with Pim2 expression in tumor-derived B cells as well as their primary cell counterparts, low expression of TRAF3 increased susceptibility to Pim inhibition in human malignant B cells, in contrast to resistance to Pim inhibitors in primary B cells. This is consistent with ‘oncogene addiction’, wherein tumors can develop dependence upon abnormally elevated proteins that promote growth and survival^42^. Oncogene addiction has been demonstrated previously with the oncogenes c-Myc and BCR-ABL in leukemia^56^. TRAF3-deficient tumors may be more likely to become dependent on Pim2 activity than TRAF3-sufficient tumors. Thus, inhibiting Pim2 may be a strategy to selectively target TRAF3-deficient B cell tumors. The Pim inhibitor SGI-1776 failed in Phase I clinical trials due to a narrow therapeutic window caused by cardiac toxicity^19^. Here, we show that the addition of c-Myc inhibitors enhanced lower-dose SGI-1776-mediated killing of TRAF3^−/−^ B cells; in the case of 10058-F4, even the lowest dose of SGI-1776 effectively killed 70–90% of TRAF3-deficient B cells when the two drugs were combined. Combined targeting of these two proteins may thus have beneficial effect in treatment of B cell malignancies deficient in TRAF3.

In both mouse and human B cells, loss of TRAF3 leads to an enhanced survival phenotype. The frequent association of *TRAF3* loss in MM and BCL, as well as mechanisms of post-translational loss of TRAF3 that may impact an additional group of B cell malignancies, suggest that this loss predisposes B cells to transformation, oncogenesis, and possibly resistance to conventional chemotherapy, because TRAF3 deficiency selectively enhances B cell survival without altering proliferation^4^. Thus, agents of conventional chemotherapy, many of which enhance DNA damage, will not kill TRAF3-deficient B cells, but could leave them with additional transforming mutations. It is thus important to fully understand the multiple ways in which TRAF3 regulates B cell survival and how these pathways may interact to most effectively treat B cell malignancies.

## Materials and Methods

### Mice

*Traf3*^flox/flox^ mice bred with *Cd19*-Cre (B-*Traf3*^−/−^) mice and extensively backcrossed onto the C57BL/6 background were previously described^4^. *Traf3*^flox/flox^ mice bred with *Cd4*-Cre (B-*Traf3*^−/−^) mice and extensively backcrossed onto the C57BL/6 background were previously described^14^. *Map14k*^−/−^ (also known as *Nik*^−/−^) mice were originally generated by Dr. Robert Schreiber (Washington University, St. Louis, MO)^57^ and provided by Dr. David Parker (Oregon Health Science University, Portland, OR). Mice of 2–4 months of age were used for all experiments. All mice were maintained under specific pathogen-free conditions and were used in accordance with National Institute of Health guidelines under an animal protocol approved by the Animal Care and Use Committee at the University of Iowa. Similar numbers of male and female mice were used in experiments presented here.

### Mouse primary cell isolation and culture

Splenic B cells were purified by negative selection as previously reported^4^. Briefly, B cells were isolated by anti-CD43 Ab-mediated negative selection, using a magnetic bead kit (Miltenyi Biotec, Auburn, CA) or mouse B cell isolation kit (STEMCELL, Vancouver, Canada), according to the manufacturers’ protocols. Mouse splenic pan-T cells were purified by negative selection with a mouse T cell isolation kit (STEMCELL). Cells, were maintained in RPMI 1640 medium (Life Technologies, Grand Island, NY) containing 10 μM 2-β-mercaptoethanol (Sigma Aldrich, St. Louis, MO), 10% heat-inactivated FBS (Atlanta Biologicals, Atlanta, GA, USA), 2 mM L-glutamine (Life Technologies), and 100 U/ml penicillin-streptomycin antibiotics (Life Technologies). This is referred to as B cell medium (BCM10).

### Cell lines

All cell lines were maintained in BCM10. Human MM-derived cell lines included OPM2^58^, LP1^59^, and RPMI8226^60^. Human DLBCL lines used were BJAB^61^ and OCI-Ly7^62^. The mouse B cell lymphoma line A20.2J^63^ was used in experiments in the supplemental material.

### Antibodies and reagents

Antibodies (Abs) used in immunoblotting included anti-phospho-BAD (S112), anti-BAD, anti-phospho-p70S6K (T389), anti-p70S6K, anti-4E-BP1 (T37/46), anti-4E-BP1, and anti-c-Myc, and were purchased from Cell Signaling Technology (Danvers, MA). Anti-TRAF3 (H122) Ab was from Santa Cruz Biotechnology (Dallas, TX) and the anti-mouse actin Ab was from Sigma (St. Louis, MO). The anti-Pim2 and anti-β-actin Abs were from Thermo Fisher (Waltham, MA). Rabbit anti-K48 polyubiquitin Ab was purchased from Abcam (Cambridge, MA). HRP-conjugated goat-anti-mouse IgG, and goat-anti-rabbit Ig Abs were from Jackson ImmunoResearch Laboratories (West Grove, PA). Cucurbatacin I was purchased from Santa Cruz Biotechnology. SGI-1776 was graciously provided by Dr. David Bearss (University of Utah, Salt Lake City, UT) or purchased from ApexBio (Houston, TX). TP-3654 was also a gift from Dr. Bearss. 10058-F4 was from Tocris (Bristol, UK). JQ1 was purchased from Cayman Chemical (Ann Arbor, MI).

### Viability assays

Splenic B cells were cultured at 0.5–1 × 10^6^ per ml. Cells were stained with the DNA dye propidium iodide (PI) and the % live cells was determined by the % of the PI-negative population, analyzed by fluorescence detection, using the Accuri C6 Flow Cytometer (BD Bioscience, San Jose, CA). The data were analyzed with FlowJo software (FlowJo LLC, Ashland, OR).

### Real-time PCR (RT-PCR)

RNA from mouse primary B cells was extracted with an RNeasy extraction kit (Qiagen, Gaithersburg, MD) and cDNA synthesized using SuperScript III polymerase (Invitrogen, Carlsbad, CA). RT-PCR was performed on an ABI PRISM 7900 Sequence Detection System (Applied Biosystems, Grand Island, NY). Primers were purchased from Applied Biosystems (Mm00454579_m1 for Pim2, Mm00487804_m1 for c-Myc, Mm00435712_m1 for Pim1, Mm01308047_g1for Pim3, and Mm99999915_g1 for GAPDH). Expression data were analyzed using the comparative Ct method^64^ and normalized to GAPDH expression.

### Immunoprecipitation

20–30 × 10^6^ cells/sample were lysed with IP Lite lysis buffer (0.5% Triton X, 40 mM Tris, 100 mM NaCl, 1 mM CaCl_2_) containing EDTA-free Mini Complete protease inhibitor (Roche) and 100 ug/mL DNase I (Roche). For assaying K48 ubiquitination, lysis buffer was supplemented with 20 mM N-ethylmaleimide (Sigma). Lysates were incubated with an Ab against c-Myc overnight at 4 °C with constant agitation. The immune complex was precipitated with protein G Dynabeads (Life Technologies), washed and resuspended in SDS-PAGE loading buffer and heated to 95 °C for 10 min.

### Transfection of mouse B cells with siRNA

B cells were transfected using electroporation following a protocol previously described for primary T cells^65^. The Pim2 and STAT3 siRNAs were purchased from Santa Cruz Biotechnology. 5–10 × 10^6^ cells were resuspended in 100 μL 2S electroporation buffer (5 mM KCl, 15 mM MgCl_2_, 15 mM HEPES, 150 mM Na_2_HPO_4_/NaH_2_PO_4_ pH 7.2 and 50 mM sodium succinate). 300 pmol of siRNA was added and cells were electroporated using the Nucleofector II electroporation system (Lonza, Basel, Switzerland), with the X-001 program. Cells were resuspended in 20% FBS-supplemented BCM without antibiotics and analyzed 48 or 24 hrs later by WB.

### Statistical analysis

Unpaired, two-tailed student’s t tests were used to evaluate differences for statistical significance for most experiments. A paired two-tailed student’s t test was used to evaluate statistical significance for paired sets of samples. An unpaired t test with Welch’s correction was to compare groups with unequal variances. The Mann-Whitney test was used for independent samples that are not normally distributed. A Wilcoxon signed rank test was used to assess statistical significance for paired samples that were not normally distributed. Statistical details are listed in the caption of each figure. Statistical analysis and graphs were prepared using GraphPad Prism (GraphPad Software, San Diego, CA). (NS = not significant, *p < 0.05, **p < 0.01, ***p < 0.001, ****p < 0.0001).

## Supplementary information

Supplementary Information
