# Author Correction: TRAF3 regulates the oncogenic proteins Pim2 and c-Myc to restrain survival in normal and malignant B cells

**DOI:** 10.1038/s41598-019-53669-2

**Published:** 2019-11-20

**Authors:** Amy L. Whillock, Nurbek Mambetsariev, Wai W. Lin, Laura L. Stunz, Gail A. Bishop

**Affiliations:** 1 Department of Microbiology & Immunology, University of Iowa, Iowa City, IA USA; 2 Internal Medicine, University of Iowa, Iowa City, IA USA; 3 Immunology Graduate Program, University of Iowa, Iowa City, IA USA; 4 Medical Scientist Training Program, University of Iowa, Iowa City, IA USA; 5 Holden Comprehensive Cancer Center, University of Iowa, Iowa City, IA USA; 6 VA Medical Center, Iowa City, IA USA; 7 Present Address: Sanford-Burnham-Prebys Medical Discovery Institute, La Jolla, CA USA; 8 Present Address: Northwestern Memorial Hospital, Chicago, IL USA

Correction to: *Scientific Reports* 10.1038/s41598-019-49390-9, published online 09 September 2019

This Article contains errors in the Introduction.

“TRAF3-deficient B cells were more susceptible to biochemical Pim2 inhibition and more resistant to inhibitors of the phosphatidyl inositol-3 kinase/protein kinase B (PI3K/Akt) pathway compared to wild type (WT) B cells. Low TRAF3 expression in human BCL and MM cell lines correlated with higher Pim2 protein levels and decreased susceptibility to Pim2 inhibitor-mediated cell death.”

should read:

“TRAF3-deficient B cells were less susceptible to biochemical Pim2 inhibition compared to wild type (WT) B cells. Low TRAF3 expression in human BCL and MM cell lines correlated with higher Pim2 protein levels and increased susceptibility to Pim2 inhibitor-mediated cell death.”

