# Supplementary material for: TRAF3 regulates the oncogenic proteins Pim2 and c-Myc to restrain survival in normal and malignant B cells
Source: Sci Rep. 2019 Sep 9;9:12884. doi: 10.1038/s41598-019-49390-9 (PMC6733949; doi:10.1038/s41598-019-49390-9)

Supplementary Material

TRAF3 regulates the oncogenic proteins Pim2 and c-Myc to restrain survival in normal and malignant B cells

Amy L. Whillock[1,3,4,5], Nurbek Mambetsariev[1,3,4#], Wai W. Lin[1,3+], Laura L. Stunz[1,5], and Gail A. Bishop[*, 1,2,3,4,5,6]

Dept. of Microbiology & Immunology[1], Internal Medicine[2], Immunology Graduate Program[3], Medical Scientist Training Program[4] and Holden Comprehensive Cancer Center[5], University of Iowa and VA Medical Center[6], Iowa City, IA

[*]**Corresponding author:**

Gail Bishop

2296 CBRB, The University of Iowa

Iowa City, IA 52242

Phone: 319-335-7945

FAX: 319-335-9006

Email: gail-bishop@uiowa.edu

[+]Present address: Sanford-Burnham-Prebys Medical Discovery Institute, La Jolla, CA

[#]Present address: Northwestern Memorial Hospital, Chicago, IL

Supplemental Fig. 1. Pim1 and Pim3 expression in TRAF3-deficient mouse B cells. A. Pim1 and Pim3 mRNA levels in WT and TRAF3$^{-/-}$ B cells were assayed with RT-PCR. Data were normalized to GAPDH and fold change was determined using the comparative Ct method. Graph depicts mean values ± SEM (N = 3 mice). An unpaired t test was used to evaluate differences for statistical significance (NS = not significant). **B.** WCLs of WT and TRAF3$^{-/-}$ B cells were analyzed with WB for Pim1 and Pim3 protein expression. WCL from the mouse B cell lymphoma cell line A20.2J was included as a positive control. Each lane represents an independent replicate (N =4).

Supplemental Fig. 2. Pim2 expression in TRAF3-deficient mouse T cells. Whole cell lysates of WT and TRAF3$^{-/-}$ T cells were analyzed by WB for Pim2, c-Myc, and β-actin expression. **A.** A representative blot of 5 independent replicates is shown. **B.** Graph depicts mean value ± SEM (N = 5 mice) of Pim2 expression. **C.** Graph depicts mean value ± SEM (N = 5 mice) of c-Myc expression. An unpaired t test was used to evaluate differences for statistical significance (NS = not significant).

Supplemental Fig. 3. Impact of loss of NIK on Pim2 expression in TRAF3-deficient mouse B cells. Whole cell lysates of WT, TRAF3$^{-/-}$, and TRAF3$^{-/-}$NIK$^{-/-}$ B cells were analyzed with WB for Pim2 and Actin expression. A representative blot from three independent experiments is shown. This figure was presented in the doctoral dissertation of N.M. [23].

Supplemental Fig. 4. Uncropped Western blot images. Western blots from figures 1-4 and supplementary figures 1-3 are provided in full-length.

Supplemental Figure S1

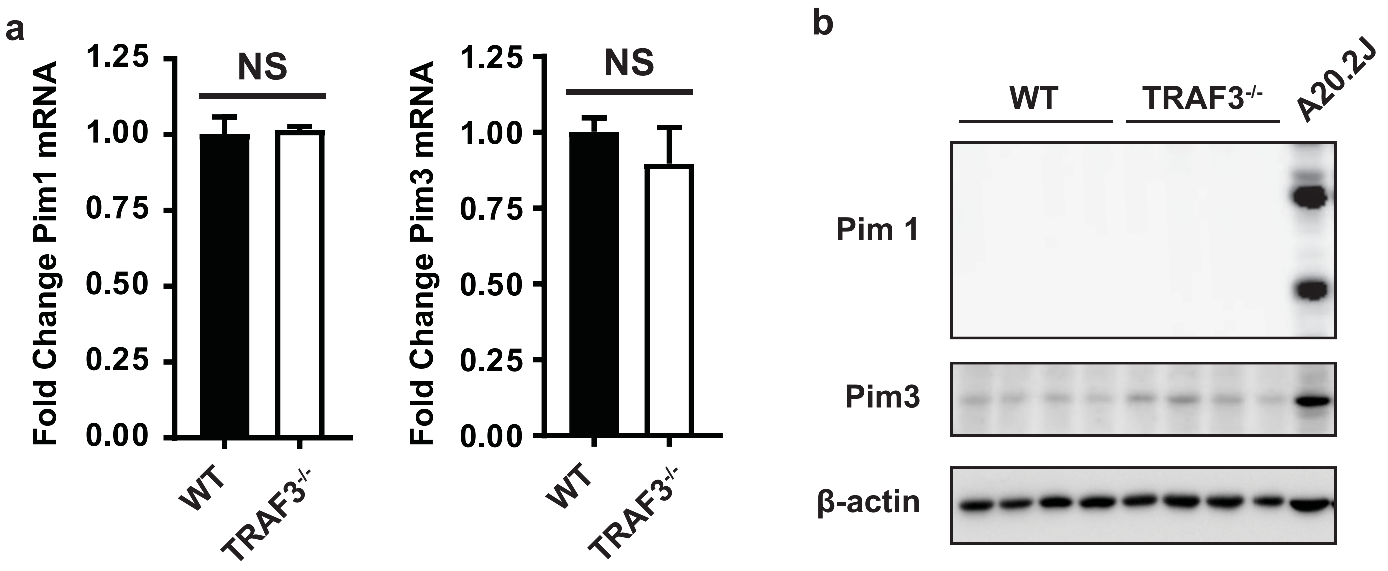

Supplemental Figure S2

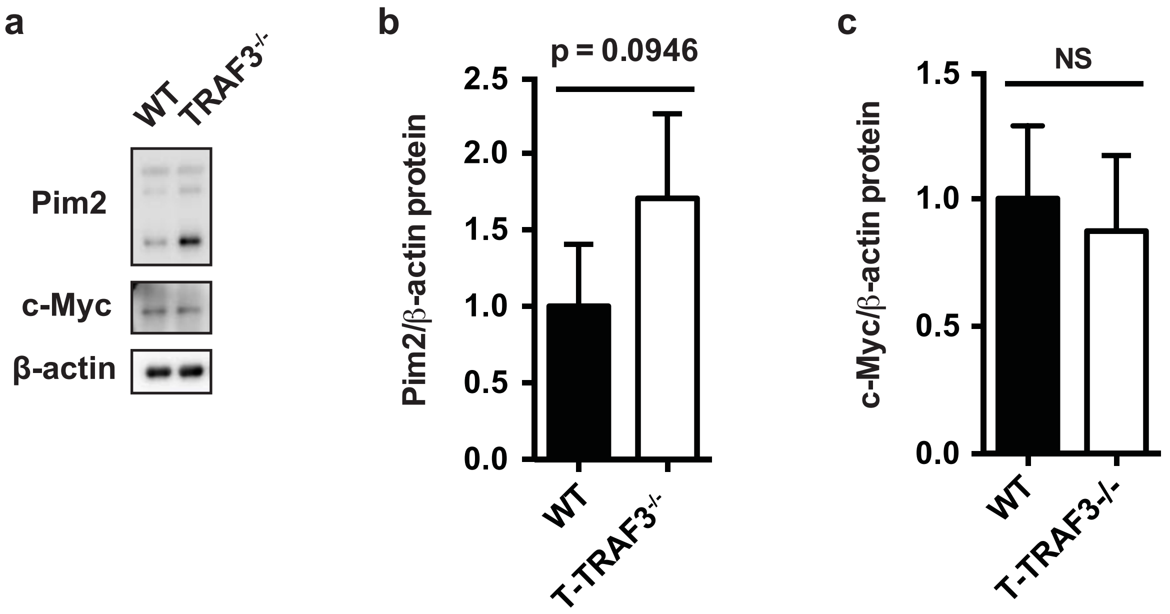

Supplemental Figure S3

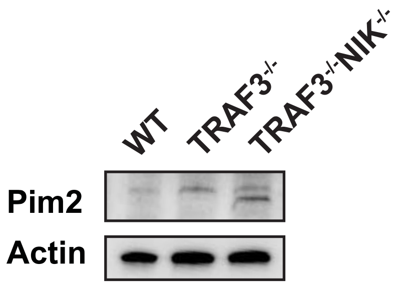

Supplemental Figure S4

Fig. 1 uncropped blots

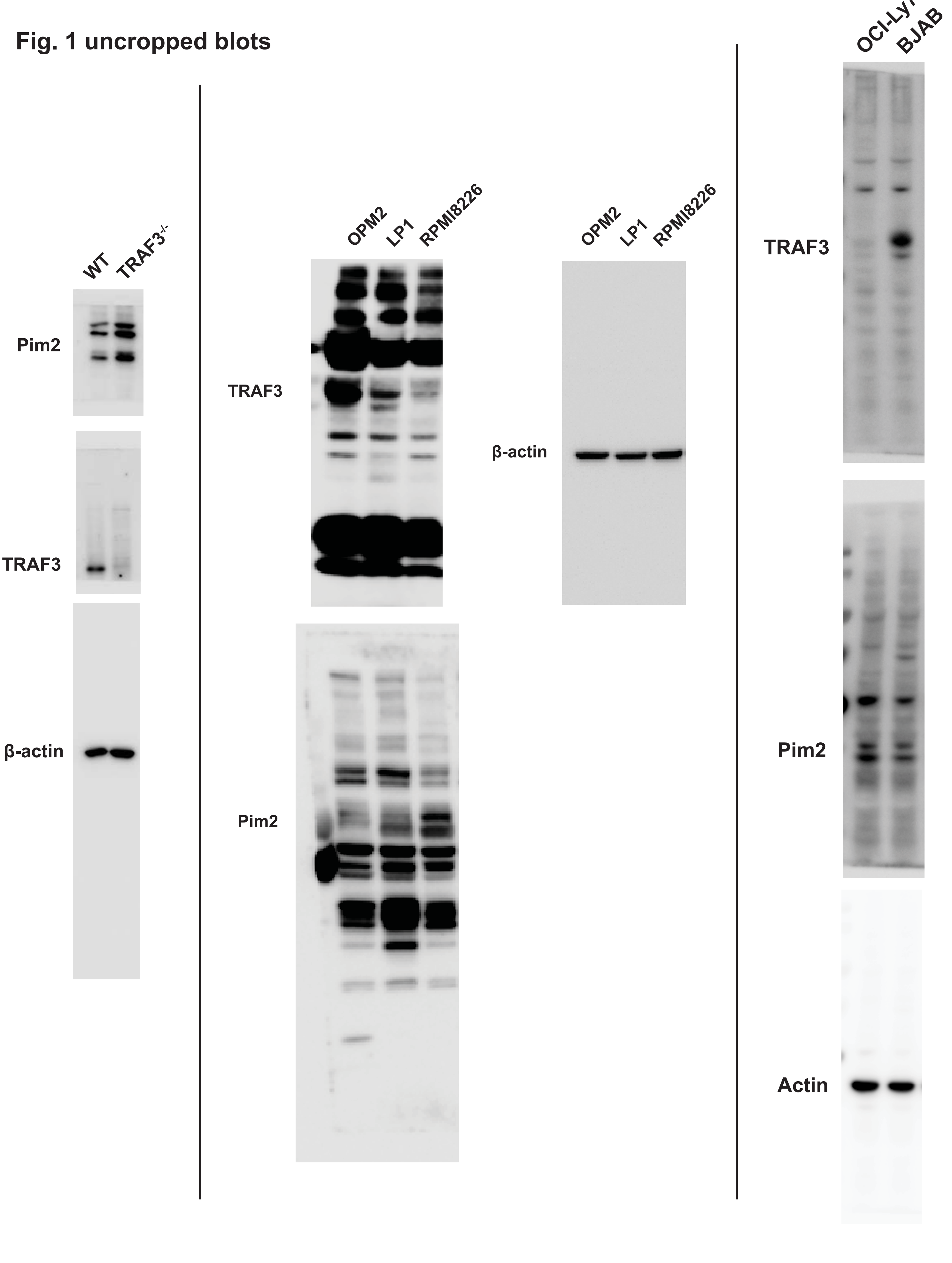

Supplemental Figure S4 Cont.

Fig. 2 uncropped blots

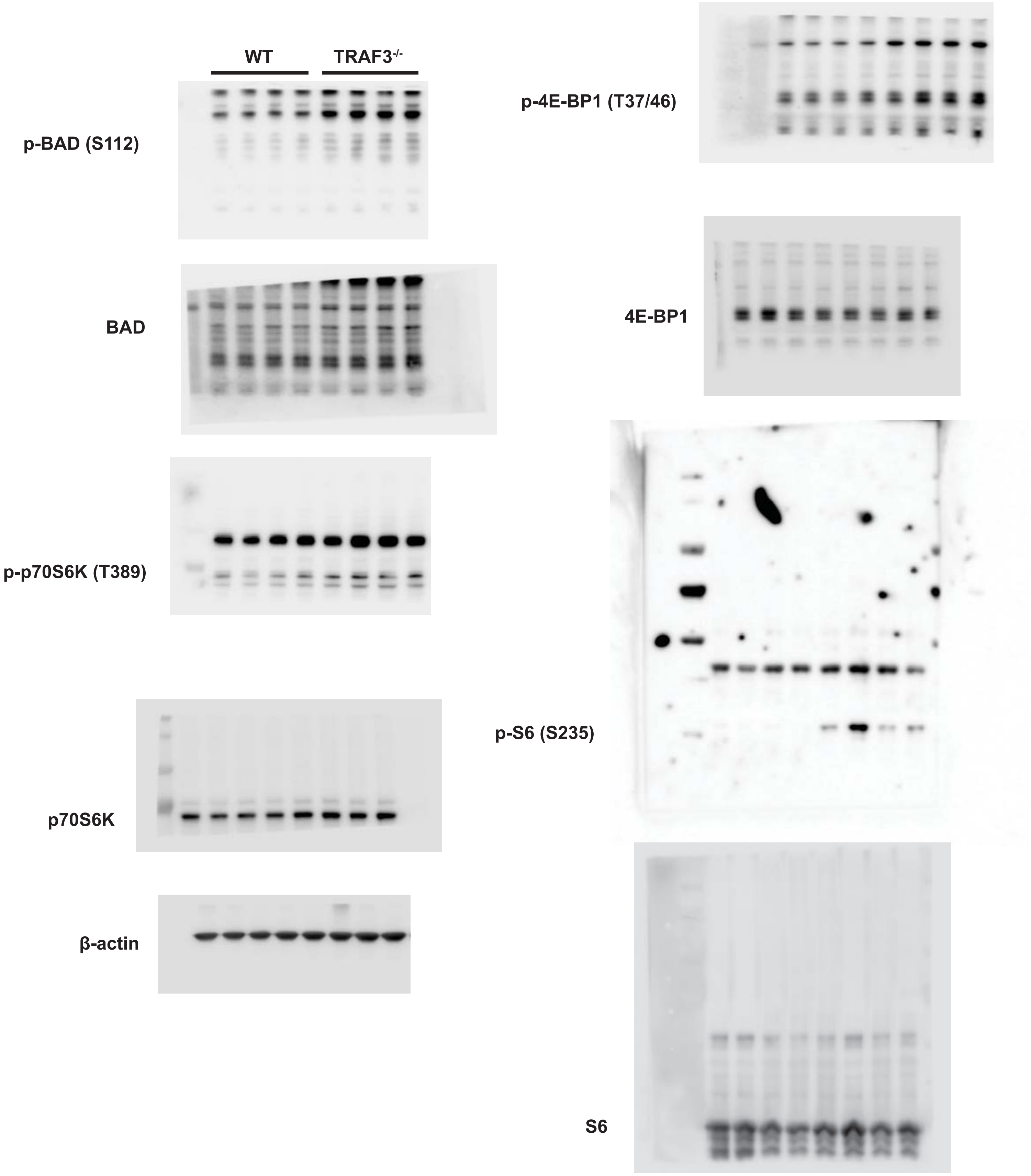

Supplemental Figure S4 Cont.

Fig. 3 uncropped blots

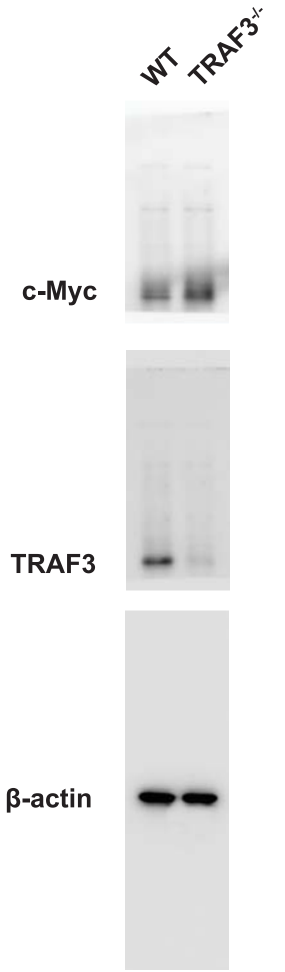

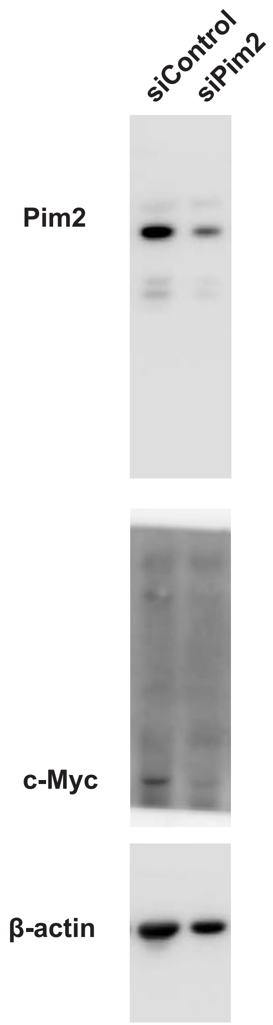

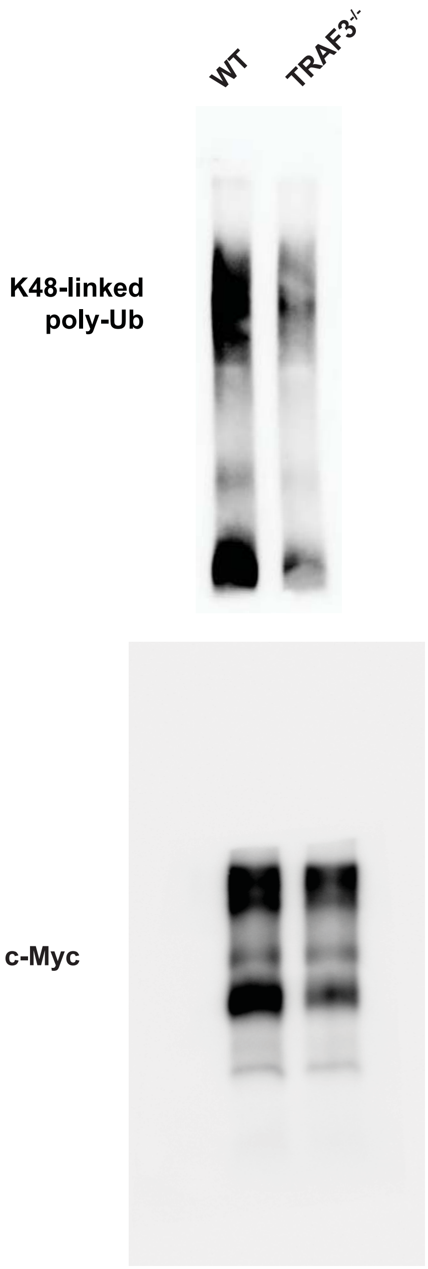

Supplemental Figure S4 Cont.

Fig. 4 uncropped blots

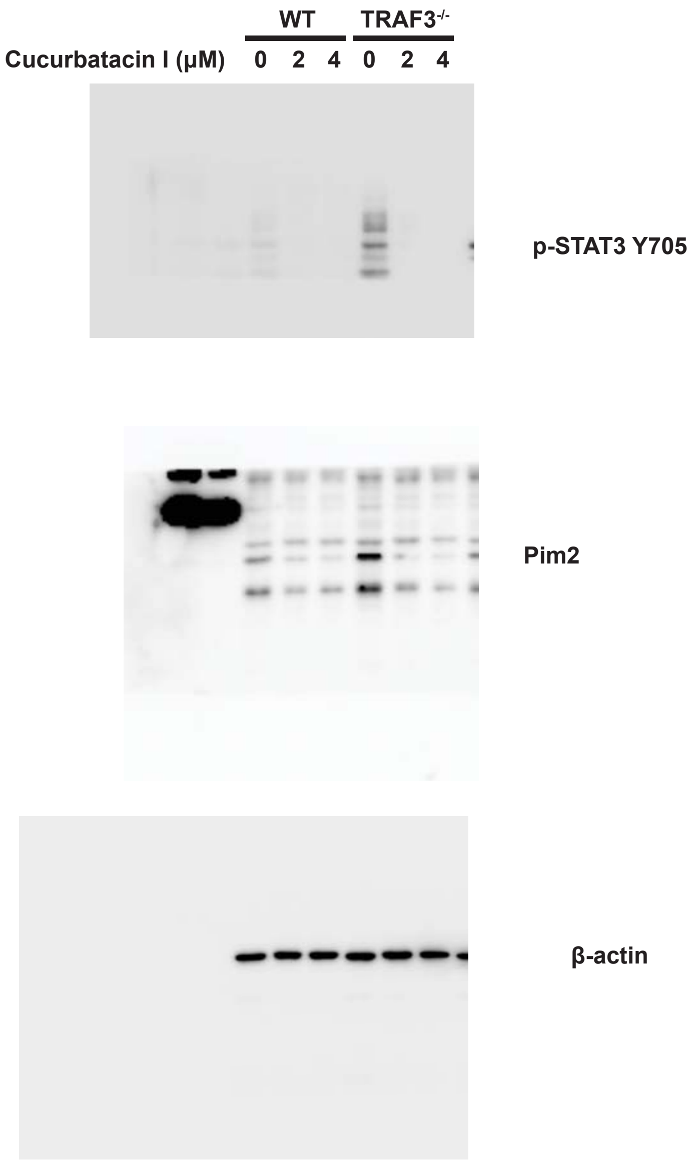

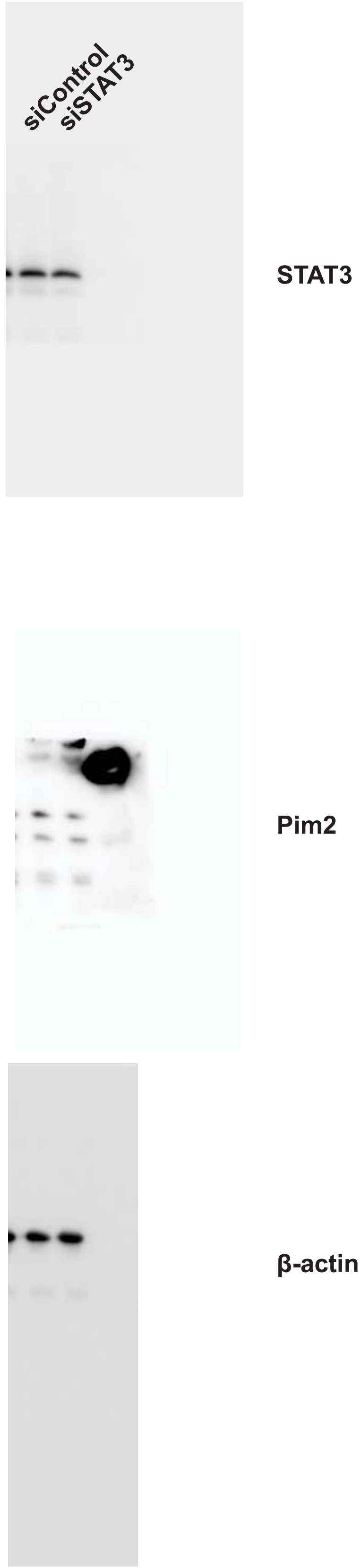

Supplemental Figure S4 Cont.

Fig. S1 uncropped blots

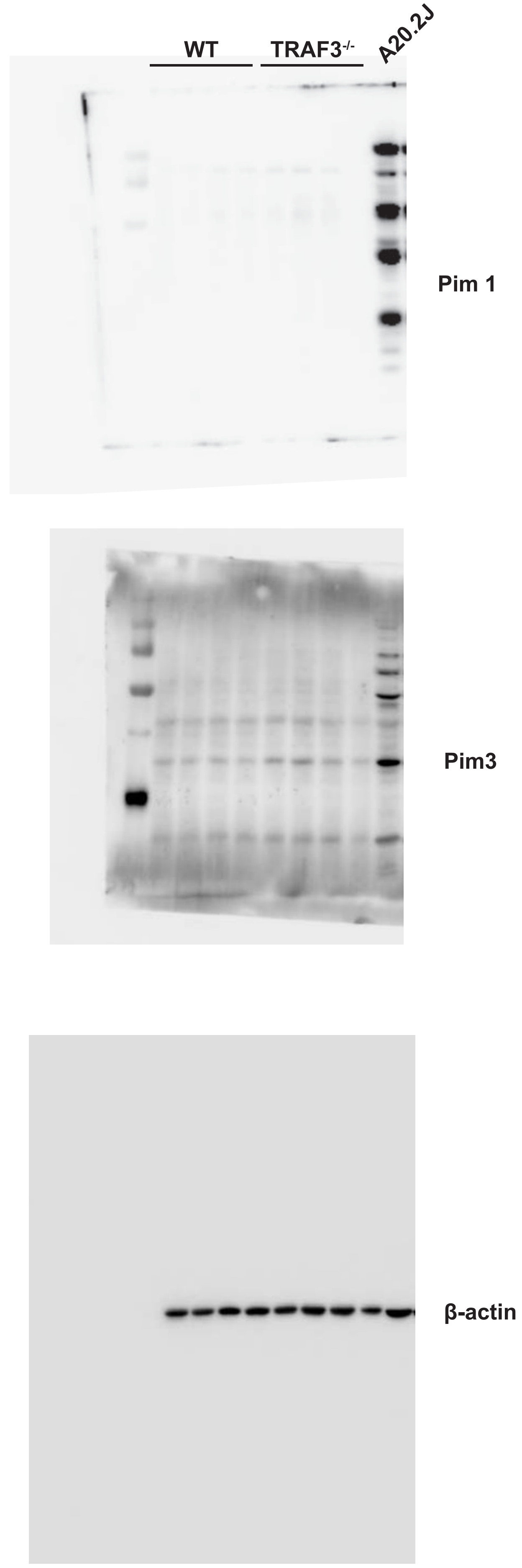

Supplemental Figure S4 Cont.

Fig. S2 uncropped blots

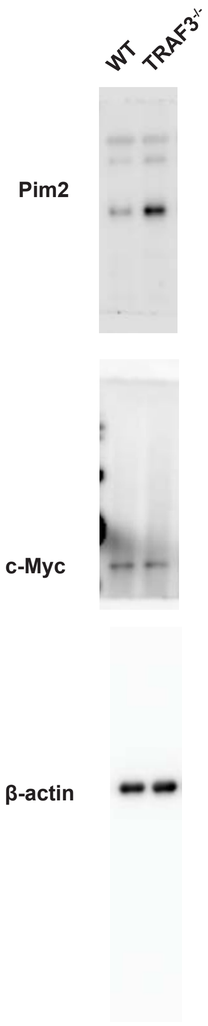

Supplemental Figure S4 Cont.

Fig. S3 uncropped blots

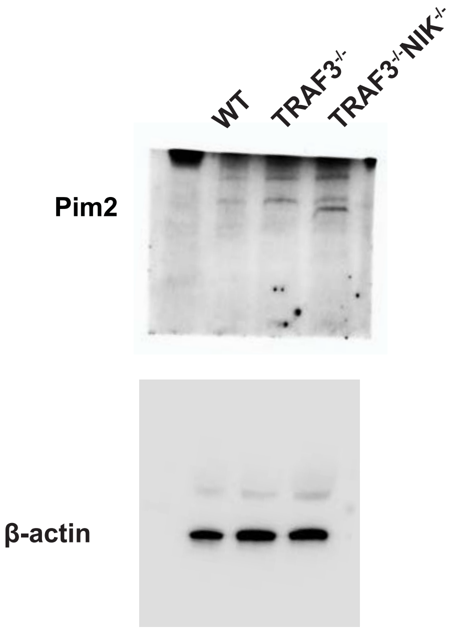

Supplement: Supplementary file 1 — Supplementary Information [file 41598_2019_49390_MOESM1_ESM.pdf]
